# Timing of initiation of breastfeeding and its determinants at a tertiary hospital in Ghana: a cross-sectional study

**DOI:** 10.1186/s12884-021-03943-x

**Published:** 2021-06-30

**Authors:** Adwoa Pokua Boakye-Yiadom, Samuel Blay Nguah, Emmanuel Ameyaw, Anthony Enimil, Priscilla Naa Lomle Wobil, Gyikua Plange-Rhule

**Affiliations:** 1 Komfo Anokye Teaching Hospital, Kumasi, Ghana; 2 Kwame Nkrumah University of Science and Technology, Kumasi, Ghana

**Keywords:** Breastfeeding initiation, Neonates, Determinants, early infant breastfeeding

## Abstract

**Background:**

Early initiation of breastfeeding (EIBF), breastfeeding within first hour after birth, is known to have major benefits for both the mother and newborn. EIBF rates, however, tends to vary between and within countries. This study set out to determine the prevalence of EIBF at the Komfo Anokye Teaching Hospital (KATH), Kumasi, Ghana, and to evaluate the determinants of EIBF and time to initiation of breastfeeding.

**Methods:**

A cross-sectional study was conducted at the KATH postnatal wards between August and October 2014. Three hundred and eighty-two mothers delivering at KATH were recruited and data on time to initiation of breastfeeding, antenatal, delivery and immediate postnatal periods were collected. Data analyses using both binary and ordinal logistic regressions with stepwise elimination were used to determine the relationship between EIBF and time to initiation of breastfeeding on one side, and the maternal, pregnancy, delivery and neonatal associated factors.

**Results:**

EIBF was done in 39.4% (95%CI: 34.3–44.5) of the newborns with breastfeeding initiated between 1 to 6 h for 19.7%, 6 to 11 h in 4.8%, 11 to 16 h in 4.8% and after 16 h in 28.5% of the deliveries. A higher number of antenatal care visits (AOR = 1.14, 95%CI: 1.04–1.25, *p* = 0.006), delivery by caesarean section (AOR = 0.07, 95%CI: 0.01–0.79, *p* = 0.031) and infant rooming-in with mother (AOR: 31.67, 95%CI: 5.59–179.43, *p* <  0.001) were significantly and independently associated with EIBF. Factors independently associated with longer time to initiation of breastfeeding were older maternal age (AOR = 1.04, 95%CI: 1.00–1.09, *p* = 0.039), Akan ethnicity (AOR = 1.92, 95%CI: 1.14–3.22, *p* = 0.014), first-born child (AOR = 2.06, 95%CI: 1.18–3.58, *p* = 0.011), mother rooming-in with newborn (AOR = 0.01. 95%CI: 0.00–0.02, *p* <  0.001), increasing fifth minute APGAR score (AOR = 0.73, 95%CI: 0.58–0.93, *p* = 0.010) and using prelacteals (AOR = 2.42, 95%CI: 1.34–4.40, *p* = 0.004).

**Conclusions:**

The low EIBF rate and prolonged time to initiation of breastfeeding at a major tertiary health facility is a major concern. Key interventions will need to be implemented at KATH and possibly other tertiary healthcare facilities in Ghana and beyond to improve EIBF rate and time to breastfeeding.

**Supplementary Information:**

The online version contains supplementary material available at 10.1186/s12884-021-03943-x.

## Introduction

Breastfeeding is the ideal way of feeding the newborn in the first six months of life for optimal growth and development [1–3]. The World Health Organisation (WHO) recommends that breastfeeding be commenced within the first hour of life, continued exclusively till six months of age when appropriate complementary feeds are added till the child is two completed years old [4]. This recommendation however has had numerous challenges in both developed and underdeveloped countries. Pain, discomfort and physical vulnerability are considered major reasons why mothers in developed countries delay or refuse to breastfeed [5, 6]. In developing countries such as Ghana, the reasons are largely different [7, 8]. Cultural beliefs about breastmilk especially colostrum, the embellishment of prelacteal feeding as well as certain food preferences are largely the reasons why breastfeeding recommendations are not followed. These cultural beliefs include colostrum being “dirty”, its role being to pave way for the real milk and thus should to be discarded. Also, water has to be given to newborns as a way of saying “welcome”, a traditional way of welcoming visitors in Ghana. Finally, prelacteals and complementary foods are believed to make children stronger [9].

Early Initiation of Breastfeeding (EIBF), the practice of putting the newborn baby to the mothers’ breast to feed within one hour of delivery has been recommended by many international agencies including the WHO. Not breastfeeding the infant within an hour but before 24 h of birth increases the risk of mortality by 40%, while delaying it till after 24 h increases it by 80% [10]. Despite these documented benefits, the prevalence of EIBF still remains low worldwide with only 57.6% of babies delivered worldwide receiving EIBF. This prevalence, however, differs widely from country to county, and region to region. In Africa it can range from as low as 34.7% in Nigeria [11] to as high as 98.4% in Angola [12]. These differing prevalence are undoubtedly due to differences in governmental enacted policies, implementation of these policies and other sociocultural practices.

Since the commencement of the recommendation of EIBF, there have been studies aimed at determining the bottlenecks that exist in its implementation. In their secondary analysis of the WHO global survey data done across three continents, Takahashi et al. [12] determined that having a caesarean delivery, developing complications during pregnancy and the absence of clearly written out postnatal and or neonatal guidelines at a hospital may affect the initiation of EIBF. Within countries however, determinants vary. In Nigeria for instance, maternal factors such as multiparity, delivery in a healthcare facility, vaginal delivery, large birth weight of the infant, higher socioeconomic status and coming from certain regions of the country were independently associated with EIBF [11]. These predictors tend to vary but are often not significantly different from region to region. An analysis of the Bangladesh Demographic and Health Survey [13] for instance revealed infant’s weight, socioeconomic status, place of delivery and place of residence were not significant predictors of EIBF.

In Ghana, as in many other African countries, there exists direct evidence that exclusive breastfeeding and EIBF saves newborn lives. Karen et al. [14] observed a dose response relationship between increased risk of neonatal death and delay in initiation of breastfeeding for one hour to seven days of life. Ghana, like many other countries, has adapted EIBF as a governmental policy for over two decades now. The EIBF rate however continues to be low despite it showing a steady increase from 46% in 2003, 52% in 2008, to 56% in 2014 [15]. The current status of EIBF in Ghana, like other countries is as a result of a mixture of home and in-hospital deliveries. Evidence shows that 73% of births in Ghana occur in a hospital, with this proportions being 90% for the urban and 59% for the rural settings [15]. Despite the obvious high proportion of babies born in hospitals there exist no documented study detailing the prevalence of EIBF and its determinants in hospital born babies specifically. This study focused on this knowledge gap in a tertiary hospital in Ghana. It further sought to determine both maternal and neonatal factors that are significantly associated with time to initiation of breastfeeding.

## Methods

### Study design

A cross-sectional study conducted at the Maternity Unit and Mother Baby Unit of the Komfo Anokye Teaching Hospital (KATH), a tertiary hospital in Kumasi, Ghana. The study population included newly delivered mothers who had live births and whose infants were alive after 24 h.

### Study site

The study was conducted in two Directorates of KATH, the Obstetrics and Gynaecology (O&G) Directorates and the Child Health Directorate. KATH is 1200 bed capacity tertiary health facility and the second largest hospital in Ghana. It is located in the second largest city in Ghana, Kumasi [16].

Kumasi is the capital of the Ashanti region, the most populous region and second largest city in Ghana. It is a typical urban city with bustling commercial activities. Traditional and cultural values are held in high esteem with a typical household often encompassing extended family members. These family members often have a huge say in how newborn infants are treated, including mode and type of feeding. Like most other places in Ghana, use of antenatal care (ANC) facilities is very high among residents of the city [15].

KATH provides Primary Health Care Services in addition to secondary and tertiary care services. It receives referrals from other hospitals and clinics in the Ashanti and surrounding regions in the middle and northern parts of Ghana. The O&G Directorate has three (3) main labour wards where about 8000 deliveries are carried out every year, with 34% of them being delivered by caesarean section. (KATH Performance Review Report 2019 – Unpublished) The O&G Directorate has three postnatal wards where newly delivered mothers with their babies are sent to after delivery. These postnatal wards can accommodate about 300 mother baby pairs and are manned by midwives with special oversight by doctors. The directorate also has a nursery where babies born to mothers who are unwell to room in with their babies are kept and nursed by midwives.

In the Child Health Directorate the study was done at the Mother and Baby Unit (MBU). MBU serves as a referral centre for sick newborns from the O&G Directorate and other surrounding health facilities. The MBU is also divided into three units: A High Dependency Unit which caters for newborn referrals that need special care; a Low Birth Weight Unit where all stable LBW infants are kept and given standard health care and a Septic Unit which admits babies up a month of age referred from the outpatient department of KATH and other hospitals. The unit admits about 4500 newborns every year and has a mortality rate of approximately 13%. (KATH Performance Review Report 2019 – Unpublished).

### Sample size

Two factors were considered in determining the sample size for the study. First was the determination of proportion of deliveries that received EIBF. This was estimated to be 59% based on the national average. Using a precision of 0.075 from the estimate and a confidence limit of 0.95, a minimum of 165 infant mother pairs was required for the study. Secondly, the sample size was determined taking into cognisance of the determinant of EIBF. To determine a minimum difference in proportion of 0.15% of the determinants, using a power of 80%, a type I error rate of 5%, and prevalence of EIBF 59%, 370 mother infant pairs were to be recruited. Thus the maximum of the two, 370 was considered the optimum sample size for the study.

### Study population

All mothers who delivered at KATH were eligible to be included in the study. However, to be included both baby and mother must be alive after 24 h and the mother must consent to being included in the study. Due to the peculiarities of the preterm infant, it was decided that the mothers who delivered before 34 completed weeks gestation as determined by a second trimester ultrasound estimation of gestational age be excluded.

### Study procedure

Between August and October 2014, all mothers who delivered at KATH were identified soon after delivery through the hospital’s inpatient registry. A systematic random sampling technique was used to select six mothers a day from the approximately 20 deliveries. The mothers were then screened using the inclusion and exclusion criteria. Those who fulfilled all the inclusion criteria and none of the exclusion criteria were approached for consenting after the study procedure has been explained to them. Most of the mothers readily consented to being included in the study but for the rare cases, two in all, where consent was not given, the next mother in the list was approached, screened and consent requested if eligible.

After consenting, a pretested structured questionnaire (please refer to the supplementary files) was administered by face-to-face interviewing of the mothers. The questionnaire was structured into four main parts.

The first part asked about the sociodemographics of the mother. These included the completed age in years, religious affiliation, marital status categorised as single or married, place of residence categorised as rural or urban, highest level of education attained and occupation, categorised as employed or unemployed.

The second section of the questionnaire asked about pregnancy and antenatal factors. These included the number of ANC visits attended before delivery, any medical condition during ANC, parity, mode of delivery and occurrence of postpartum complications. These responses from the mothers were corroborated with that recorded in the maternal and child health record book and their respective medical records. For mothers who delivered via caesarean section the type of anaesthesia given was also recorded.

The third part of the questionnaire asked about neonatal factors including the birth order of the current baby, mother practicing skin-to-skin after delivery and ward on which baby is placed after birth. The ward was recorded as either the baby rooming-in with the mother, sent to the nursery or to the MBU. Other neonatal factors such as the birth weight, which was recorded to one decimal place, the first- and fifth-minute APGAR scores (a score used to rapidly determine the health of newborns at birth) were obtained from the birth records of the child as well as the maternal and child health record book.

The final section of the questionnaire was directed towards the breastfeeding history of the child. This included finding out if the mother had ever successfully breastfed, the time it took to initiate breastfeeding for the current baby and the administration of prelacteal feed. Time to initiation of breastfeeding was determined by first recording the time of delivery and time baby was put to breast for the first time. In cases where this was missing from the mother’s clinical record, it was obtained by interviewing the mothers to give an estimation of the duration between birth and first breastfeeding. For mother who underwent caesarean section, the time to initiation of breastfeeding was obtained from the mother’s medical record that usually contains the time of delivery and time baby was put to breast.

### Data management and statistical analysis

Completed questionnaires were doubly entered into a predesigned electronic database using EpiData 3.1 (Odense, Denmark). The two datasets were then compared weekly and cleaned of abnormal or wrongful entries. Upon completion of data collection, the final dataset was exported to STATA 13.1 (StataCorp LP., College Station, TX, USA) for analysis.

Time to initiation of breastfeeding was categorised into four; less than one hour, one to six hours, greater than six hours but less than 16 h and 16 h or more. The prevalence of these times was determined with their binomial exact 95% confidence intervals. Crude association between the maternal sociodemographic, obstetric history, infant characteristics, past and current feeding practices and EIBF was determined and reported initially as the crude odds ratio with their respective 95% confidence interval. To determine the best fit model for the prediction of EIBF, a backward stepwise logistic regression model with a significance level cut-off for elimination from the model of 0.2 was done with all predictors to start with. After the stepwise elimination of the least predictive dependent variables, the adjusted odd ratios with their confidence intervals were computed using all retained variables.

To determine predictors of time to initiation of breastfeeding an ordered logistic regression was fitted. This was first done for each variable separately to determine crude associations and report as odd ratio with 95% confidence intervals. Similarly, to determine the best fit model for the predictors of time to initiation of breastfeeding, a backward stepwise ordinal logistic regression model with a significance level cut-off for elimination from the model of 0.2 was done with all predictors to start with. The best fitting model was then reported. For all analysis a two sided *p*-value of < 0.05 was considered statistically significant.

## Results

In all, 382 mother-infant pairs were recruited for this study but 376 were included in the analysis. The description of the maternal, pregnancy and infant characteristics are as shown in Table 1. The mean (+SD) age of the mothers was 29 (+ 6.4) years. The majority of the mothers were from the local Akan tribe (73.1%), married (86.7%), employed (82.7%), had completed a minimum of secondary schooling (73.1%), of the Christian religious faith (83.2%) and used the national health insurance (99.5%). The median (IQR) parity was 3(1–4) and mean(+SD) number of ANC visits before delivery was 7.0(+ 3.1). Forty-four (11.7%) mothers were known to have a medical condition before delivery while 12(3.2%) had various postpartum complications. Caesarean section rate was 34.8%. For 30.3% of the mothers, the current delivery was their first while 34.8% had never breastfed. Only 27(7.2%) of the babies were less than 38 completed weeks at the time of delivery. First and fifth minute APGAR scores of three and below were seen in 16(4.3%) and 4(1.1%) of the babies respectively. Only 216 (57.5%) of the babies roomed-in with their mothers within the first 24 h while 110 (29.3%) were given prelacteal (largely infant formula).

**Table 1:** Maternal, pregnancy and infant characteristics of study participants

Maternal Characteristics			
	n (%)		n (%)
Age in years mean (SD)	29.0 (6.4)	Educational status	
Ethnicity		None	37 (9.8)
Others	101 (26.9)	Primary	21 (5.6)
Akan	275 (73.1)	Secondary	271 (72.1)
Marital Status		Tertiary	47 (12.5)
Single	50 (13.3)	Employment status	
Married	326 (86.7)	Unemployed	65 (17.3)
Residence		Employed	311 (82.7)
Rural	5 (1.33)	First Baby	
Urban	371 (98.7)	No	262 (69.7)
Insurance type		Yes	114 (30.3)
Private	2 (0.5)	Religion	
Government	374 (99.5)	Moslem	63 (16.8)
		Christian	313 (83.2)
**Pregnancy Characteristics**			
	**n (%)**		**n (%)**
Medical conditions		Mode of delivery	
No	332 (88.3)	Vaginal	245 (65.2)
Yes	44 (11.7)	Caesarean Section	131 (34.8)
Postpartum complications		Previously breastfed	
No	364 (96.8)	No	263 (69.9)
Yes	12 (3.2)	Yes	113 (30.1)
ANC visits mean (SD)	7.0 (3.1)	Parity median (IQR)	3.0 (1.0–4.0)
**Newborn Characteristics**			
	**n (%)**		**n (%)**
Maturity		Skin to Skin contact done	
Preterm	27 (7.2)	No	219 (58.2)
Term	349 (92.8)	Yes	157 (41.8)
Gender		Where baby was kept	
Female	177 (47.1)	Roomed In	216 (57.5)
Male	199 (52.9)	Nursery	94 (25.0)
Prelacteals given		MBU	66 (17.5)
No	266 (70.7)	Birth Weight (kgs) *mean (SD)*	3.0 (0.6)
Yes	110 (29.3)		
First Min. APGAR		Fifth Min. APGAR	
0–3	16 (4.3)	0–3	4 (1.1)
4–7	246 (65.4)	4–7	41 (10.9)
8–10	114 (30.3)	8–10	331 (88.0)

EIBF was done in 39.4% (95%CI: 34.3–44.5) of the newborns. The number of newborns who were initiated on breastfeeding in less than an hour, one to less than 6 h, six to less than 16 h, and 16 h or more after delivery were 148 (39.4, 95%CI: 34.5–44.4), 74 (19.7, 95%CI: 16.0–24.0), 47 (12.5, 95%CI: 9.5–16.3) and 107 (28.5, 95%CI, 24.1–33.2) respectively.

The maternal factors that were crudely associated with EIBF (Table 2) were increasing maternal age (COR = 0.96, 95%CI: 0.93–0.99) and mother being married (COR = 0.50, 95%CI: 0.28–0.92). None of the pregnancy related variables were crudely associated with EIBF, however, number of ANC visits before delivery showed a marginal clinically significant relationship with EIBF. Neonatal factors that were significantly associated with EIBF were caesarean mode of delivery (COR = 0.005, 95%CI: 0 .000–0.037), term neonate (COR = 4.04, 95%CI: 1.37–11.93), higher birth weight (COR = 1.57, 1.08–2.29), infant roomed-in with mother (COR = 106.9, 95%CI: 32.9–346.8) and higher five-minute APGAR score (COR = 1.65, 95%CI: 1.26–2.17). After stepwise removal of insignificant factors and running the final model, higher number of ANC visits (AOR = 1.14, 95%CI: 1.04–1.25, *p* = 0.006), caesarean mode of delivery (AOR = 0.07, 95%CI: 0.01–0.79, *p* = 0.031) and infant rooming-in with mother (AOR = 31.67, 95%VI: 5.59–179.43, *p* <  0.001) were the only three factors significantly associated with EIBF.

**Table 2:** Determinants of Early Infant Breastfeeding

	EIBF n(%)					
Characteristic	No	Yes	COR (95%CI)	p-value	AOR (95%CI)	p-value
Maternal age in years *mean (SD)*	29.6 (6.5)	27.9 (6.1)	0.96 (0.93–0.99)	0.013	0.95 (0.89–1.00)	0.059
Akan ethnicity	173 (75.9)	102 (68.9)	0.70 (0.44–1.19)	0.138		
Married	205 (89.9)	121 (81.8)	0.50 (0.28–0.92)	0.025		
Urban residence	225 (98.7)	146 (98.7)	0.97 (0.16–5.90)	0.977		
Government Insurance use	226 (99.1)	148 (100.0)	–	–		
Educated mother	37 (9.84)	339 (90.2)	0.84 (0.42–1.66)	0.611	0.41 (0.13–1.31)	0.134
Employed mother	193 (84.7)	118 (79.7)	0.71 (0.42–1.22)	0.219		
Christian religion	194 (85.1)	119 (80.4)	0.72 (0.42–1.24)	0.236		
First born child	73 (32.0)	41 (27.7)	0.81 (0.52–1.28)	0.374		
ANC visits done *mean (SD)*	6.8 (3.1)	7.4 (3.2)	1.07 (0.99–1.14)	0.056	1.14 (1.04–1.25)	0.006
Medical conditions in pregnancy	31 (13.6)	13 (8.8)	0.61 (0.31–1.21)	0.159		
Parity *mean (SD)*	2.7 (1.5)	2.7 (1.5)	1.01 (0.88–1.16)	0.919		
Caesarean mode of delivery	130 (57.0)	1 (0.7)	0.005 (0 .000–0.037)	< 0.001	0.07 (0.01–0.79)	0.031
Mother previously breastfed	72 (31.6)	41 (27.7)	0.83 (0.53–1.31)	0.423	0.52 (0.24–1.12)	0.097
Post-partum complication present	12 (5.3)	0 (0.0)	–	–		
Term Infant	205 (89.9)	144 (97.3)	4.04 (1.37–11.93)	0.012		
Birth Weight in kgs *mean (SD)*	3.0 (0.6)	3.1 (0.5)	1.57 (1.08–2.29)	0.018	0.12 (0.01–1.13)	0.064
Skin-to-skin contact practiced	145 (63.6)	12 (8.1)	0.051 (0.03–0.10)	< 0.001		
Roomed in with mother	71 (31.1)	145 (98.1)	106.9 (32.9–346.8)	< 0.001	31.67 (5.59–179.43)	< 0.001
Male infant	114 (50.0)	85 (57.4)	1.35 (0.89–2.05)	0.159		
First minute APGAR	8.1 (1.5)	8.6 (1.0)	1.16 (0.99–1.36)	0.068		
Fifth minute APGAR	8.1 (1.4)	8.6 (0.6)	1.65 (1.26–2.17)	< 0.001	1.47 (0.92–2.33)	0.104
Prelacteals given	110 (48.3)	0 (0.0)	–	–		

Many of the considered factors in this study had a crude association with time to initiation of breastfeeding. (Table 3) The factors with crude significant association with time to initiation of breastfeeding were maternal age, marital status, employment status of mother, presence of a known medical condition during pregnancy, mode of delivery, presence of post-partum complications, maturity of the newborn, birth weight of the newborn, skin to skin contact practice, newborn rooming-in with mother, administrations of prelacteals, the first minute and fifth minute APGAR scores. After selecting a good fit model and adjusting for all included factors, the maternal sociodemographic factors that were significantly associated with time to initiation of breastfeeding were advancing maternal age (AOR = 1.04, 95%CI: 1.00–1.09, *p* = 0.039) and coming from the local Akan ethnicity (AOR = 1.92, 95%CI: 1.14–3.22, *p* = 0.014). Pregnancy factors associated with the time to initiation of breastfeeding were neonate being the first born (AOR = 2.06, 95%CI: 1.18–3.58, *p* = 0.011). Neonatal factors associated with time to initiation of breastfeeding were rooming-in the child with the mother (AOR = 0.01. 95%CI: 0.00–0.02, *p* <  0.001) and the giving of prelacteal (AOR = 2.42, 95%CI: 1.34–4.40, *p* = 0.004) and higher five-minute APGAR scores (AOR = 0.73, 95%CI: 0.58–0.93. *p* = 0.010).

**Table 3:** Determinants of time to initiation of breastfeeding

Characteristic	COR (95%CI)	p-value	AOR (95%CI)	p-value
Maternal age in years	1.04 (1.01–1.07)	0.003	1.04 (1.00–1.09)	0.039
Akan ethnicity	1.44 (0.95–2.19)	0.087	1.92 (1.14–3.22)	0.014
Married	2.15 (1.23–3.76)	0.007		
Urban residence	0.72 (0.14–3.84)	0.701		
Government Insurance use	0.35 (0.03–4.25)	0.413		
Educated mother	1.02 (0.54–1.92)	0.961		
Employed mother	1.63 (1.00–2.64)	0.049		
Christian religion	1.56 (0.95–2.55)	0.078		
First born child	1.14 (0.77–1.70)	0.507	2.06 (1.18–3.58)	0.011
ANC visits done	0.95 (0.90–1.01)	0.120		
Medical conditions in pregnancy	2.59 (1.40–4.77)	0.002	1.91 (0.96–3.82)	0.065
Parity	0.97 (0.85–1.10)	0.612		
Caesarean section delivery	34.9 (20.4–59.5)	< 0.001		
Mother previously breastfed	1.09 (0.73–1.62)	0.675		
Post-partum complication present	3.08 (1.11–8.61)	0.031		
Term Infant	0.14 (0.06–0.33)	< 0.001		
Birth weight	0.56 (0.4–0.78)	0.001		
Skin-to-skin contact practiced	24.5 (14.9–40.2)	< 0.001		
Roomed in with mother	0.01 (0.00–0.01)	< 0.001	0.01 (0.00–0.02)	< 0.001
Male infant	0.80 (0.55–1.16)	0.240		
First minute APGAR	0.82 (0.72–0.94)	0.006		
Fifth minute APGAR	0.63 (0.52–0.76)	< 0.001	0.73 (0.58–0.93)	0.010
Prelacteals given	9.66 (6.18–15.10)	< 0.001	2.42 (1.34–4.40)	0.004

## Discussion

In this study, we set out to estimate the rate or EIBF, determinants of EIBF and determinants of time to initiation of breastfeeding in a major tertiary hospital in Ghana. The EIBF rate among babies delivered at KATH of 39.4% is quite low compared the national average of 56%. Factors that independently predicted the practice of EIBF were delivery per vagina, higher number of ANC visits by the mother before delivery and mother infant pair rooming-in together. Determinants of time to initiation of breastfeeding however included maternal age, ethnicity, birth order of the neonate, mother infant pair rooming-in together, administration of prelacteals (infant milk formulas) to the newborn and higher five-minute APGAR scores.

KATH is a major referral centre in the northern sector of Ghana, implying a higher proportion of mothers delivering at KATH would have complicated pregnancies compared the national proportion. This assertion is supported by the very high proportion of caesarean sections and mothers with pregnancy related medical conditions in our study. The structure of our study population could be the major reason for this large difference between the in-hospital and national EIBF rates. Though there is enough evidence that EIBF rates tend to vary in this manner, both within the West African sub region and beyond, [12] it is also evident that some practices within KATH may contribute significantly to the low EIBF rate. With such a low proportion of newborns rooming-in with their mothers and the high occurrence of use of prelacteal, it is not surprising that the EIBF rate is low. The Baby Friendly Hospital Initiative, [4] when adopted, is expected to promote EIBF among mothers in all hospitals. After adoption and implementation of these ten steps to becoming Baby Friendly, some high level care hospitals, even in developed countries, have markedly improved their EIBF rate [17]. To improve the EIBF status therefore requires a whole paradigm shift in managing mothers and infants before, during and after delivery.

In our study, the main determinant of EIBF was rooming-in, a major component of the Baby Friendly Hospital Initiative. However, there were other notable determinants of not receiving EIBF namely; caesarean mode of delivery and lower number of ANC visits before delivery. It is of little doubt that both physical and psychological trauma associated with caesarean delivery before, during and after pregnancy can be a big disincentive for EIBF. Worldwide, caesarean section delivery has been a consistent significant independent factor associated with decreased practice of EIBF [11, 12, 18]. Complications to both the mother and newborn infants make this group a high risk group. However, KATH, and for that matter major health institutions in Ghana can improve EIBF rate if proper feeding practices are aggressively adapted by healthcare personnel after caesarean section deliveries and mothers are supported enough by the midwives/health staff to initiate breastfeeding early. Considering the fact that caesarean section deliveries rate in our study was approximately 35%, and all but two mothers had spinal anaesthesia, implementing interventions such as early skin to skin contact and immediate putting of the baby to the breast are likely to improve the EIBF rate significantly in many health institutions [19].

In Ghana, the nurses and midwives consistently educate prospective mothers on the need and benefits of EIBF and breastfeeding in general at every ANC visits. Therefore, it is not surprising that higher number of ANC visits in our study is associated with EIBF. This is irrespective of the fact that Ghana has adapted the WHO policy on breastfeeding with no central regulation nor specific incentives given to the mothers to breastfeed. This is indeed an observed phenomenon in many studies from places where similar messages are taught to the mothers at every ANC visit [20, 21]. Current literature however indicates a mixed conclusion concerning the association between the number of ANC visits and EIBF [13]. Studies that do not show a relationship between EIBF and number of ANC visits often reveal inconsistent or no education by healthcare persons during routine visits [22].

Inasmuch as EIBF is recommended because of its well documented benefits there will be conditions under which it might not be possible to achieve. More than a quarter (28.5%) of the newborns in our study were breastfed for the first time after 16 h. At first glance this observation could be attributed to the clinical states of the mothers, such as maternal illness before, at and after delivery as well as the infant’s clinical states after delivery including the APGAR score. However, modifiable factors such as infant rooming-in with mother and the administering of prelacteal are seen as stronger determinants of longer time to breastfeeding.

These observations underpin the fact that a greater emphasis on antenatal education on the effects and benefits of breastfeeding, an intervention with a proven success rate even in countries with very limited exclusive and breastfeeding rate must be encouraged and implemented at health institutions in Ghana. Secondly, in-hospital modifiable factors such as baby rooming-in with mother and discouraging the use of prelacteal, all of which are component of the Baby Friendly Hospital Initiative, when implemented should help reduce time to breastfeeding.

### Limitations

Despite the obvious strength of our study in term of being carried out in a large hospital with a high number of babies born, there are some notable limitations. First, the time of breastfeeding was obtained from recall by the mothers. This invariably introduces recall bias. Secondly, the clinical setting of our study may be very different from what pertains in many of other rural areas in Ghana, thus making the finding and wholesale application of the recommendations problematic in these areas. Finally, this was a single centre study therefore limiting its generalisability to other tertiary hospitals.

## Conclusion

More than a third (39.4%) of babies born at KATH benefited from EIBF but as many as 23.3% of them were breastfed after 16 h from delivery. Independent factors significantly associated with EIBF were increasing number of ANC visits, vaginal mode of delivery compared to caesarean delivery and rooming-in mother and baby. The determinants associated with longer time to initiating breastfeeding were increasing maternal age, coming from the Akan ethnic group and infant being a first born. Other significantly associated risk factors include mother and infant not rooming-in together after delivery, higher fifth minute APGAR scores and giving prelacteal feeds to the baby.

## Supplementary Information

**Additional file 1.**

## Data Availability

The datasets generated and/or analysed during the current study are available in the Mendeley repository available at https://data.mendeley.com/datasets/y9h89fvfwf/1

## References

[1] W.H.O. Exclusive breastfeeding for six months best for babies everywhere. 5th January statement. 2011;:http://www.who.int/mediacentre/news/statements/201. http://www.who.int/mediacentre/news/statements/2011/breastfeeding_20110115/en/. Accessed 30 August 2020.

[2] Langa L (2010). Breast is always best, even for HIV-positive mothers. Bull World Health Organ.

[3] WHO. WHO | Global Strategy: Breastfeeding critical for child survival. Who. 2010. https://www.who.int/mediacentre/news/releases/2004/pr19/en/. Accessed 30 August 2020.

[4] WHO. Infant and young child nutrition: Global strategy on infant and young child feeding. Fifty Fifth World Heal Assem. 2002;53 April:1–18. http://apps.who.int/gb/archive/pdf_files/WHA55/ea5515.pdf. Accessed 22 September 2020.

[5] Kelleher CM (2006). The physical challenges of early breastfeeding. Soc Sci Med.

[6] Ogbuanu CA, Probst J, Laditka SB, Liu J, Baek JD, Glover S (2009). Reasons why women do not initiate breastfeeding. A southeastern state study. Women’s Heal Issues.

[7] Aborigo RA, Moyer CA, Rominski S, Adongo P, Williams J, Logonia G, et al. Infant nutrition in the first seven days of life in rural northern Ghana. BMC Pregnancy Childbirth. 2012;12(1). 10.1186/1471-2393-12-76. 10.1186/1471-2393-12-76 PMC3490996 22857600

[8] Da Silva MC, Gunnlaugsson G, Smedman L (1992). Determinants of delayed initiation of breastfeeding: a community and hospital study from Guinea-Bissau. Int J Epidemiol.

[9] Joyce N. Socio-cultural factors influencing exclusive breastfeeding practices among women in the Bawku West District, Ghana University of Ghana. 2017. http://ugspace.ug.edu.gh.

[10] Edmond K, Newton S, Hurt L, Shannon CS, Kirkwood BR, Mazumder S (2016). Timing of initiation, patterns of breastfeeding, and infant survival: prospective analysis of pooled data from three randomised trials. Lancet Glob Heal.

[11] Berde AS, Yalcin SS (2016). Determinants of early initiation of breastfeeding in Nigeria: a population-based study using the 2013 demograhic and health survey data. BMC Pregnancy Childbirth..

[12] Takahashi K, Ganchimeg T, Ota E, Vogel JP, Souza JP, Laopaiboon M, et al. Prevalence of early initiation of breastfeeding and determinants of delayed initiation of breastfeeding: secondary analysis of the WHO global survey. Sci Rep. 2017;7(1). 10.1038/srep44868. 10.1038/srep44868 PMC5359598 28322265

[13] Karim F, Salam Khan AN, Tasnim F, Kabir Chowdhury MA, Billah SM, Karim T (2019). Prevalence and determinants of initiation of breastfeeding within one hour of birth: an analysis of the Bangladesh demographic and health survey, 2014. PLoS One.

[14] Edmond KM, Zandoh C, Quigley MA, Amenga-Etego S, Owusu-Agyei S, Kirkwood BR (2006). Delayed breastfeeding initiation increases risk of neonatal mortality. Pediatrics..

[15] GSS. 2014 Demographic and Health Survey Fact Sheet Ghana. Report. 2014;:2. http://dhsprogram.com/pubs/pdf/GF36/GF36.pdf. Accessed 31 August 2020.

[16] Komfo Anokye Teaching Hospital. Komfo Anokye Teaching Hospital | “A Centre of Excellence.” 2019. http://www.kathhsp.org/. .

[17] Pérez-Escamilla R, Martinez JL, Segura-Pérez S (2016). Impact of the baby-friendly Hospital initiative on breastfeeding and child health outcomes: a systematic review. Matern Child Nutr.

[18] John JR, Mistry SK, Kebede G, Manohar N, Arora A (2019). Determinants of early initiation of breastfeeding in Ethiopia: a population-based study using the 2016 demographic and health survey data. BMC Pregnancy Childbirth..

[19] Dudeja S, Sikka P, Jain K, Suri V, Kumar P (2018). Improving first-hour breastfeeding initiation rate after cesarean deliveries: a quality improvement study. Indian Pediatr.

[20] Vieira TO, Vieira GO, Giugliani ERJ, Mendes CM, Martins CC, Silva LR. Determinants of breastfeeding initiation within the first hour of life in a Brazilian population: cross-sectional study. BMC Public Health. 2010;10(1). 10.1186/1471-2458-10-760. 10.1186/1471-2458-10-760 PMC3020684 21143893

[21] Ahmad MO, Sughra U, Kalsoom U, Imran M, Hadi U (2012). Effect of antenatal counselling on exclusive breastfeeding. J Ayub Med Coll Abbottabad.

[22] Dhandapany G, Bethou A, Arunagirinathan A, Ananthakrishnan S (2008). Antenatal counseling on breastfeeding - is it adequate? A descriptive study from Pondicherry, India. Int Breastfeed J.

