# Supplementary material for: Timing of initiation of breastfeeding and its determinants at a tertiary hospital in Ghana: a cross-sectional study
Source: BMC Pregnancy Childbirth. 2021 Jun 30;21:468. doi: 10.1186/s12884-021-03943-x (PMC8247238; doi:10.1186/s12884-021-03943-x)
Supplement: Supplementary file 1 — Additional file 1. [file 12884_2021_3943_MOESM1_ESM.pdf]

FACTORS AFFECTING INITIATION OF BREASTFEEDING IN A TERTIARY HOSPITAL IN GHANA

Study ID	[__][__][__]
**SECTION A: Socio-demographic characteristics**	
**Mothers age in years**	[__][__]years
**Religion**<br>Christian ……………………….1<br>Traditional……………………...2<br>Moslem………………………....3<br>Others ………………………….4	[__]
**Ethnic Groups (*circle the appropriate*)**<br>**Akan --------------------------------1**<br>**Ga ---------------------------------- 2**<br>**Ewe ----------------------------------3**<br>**Ethnic groups from the north---4**<br>**Others/foreign ---------------------5**	[__]
**Marital status (0=*Married* 1=*Single*)**	[__]
**Residence (0=*Urban* 1=*Rural*)**	[__]
**Insurance type (0=*National* 1=*Private*)**	
**What is your highest level of educational**<br>None…………………………….……..0<br>Primary…………………….….…1<br>Secondary……………………….. 2<br>Tertiary…………………...……3	[__]
**Mothers Employment Status (0=*Employed* 1=*Unemployed*)**	[__]
**SECTION B: Birth History**	
**Is this your first baby (0=*No* 1=*Yes*)**	[__]
**Maturity of newborn (0=*Preterm* 1=*Term*)**	[__]
**History of skin to skin contact at birth (0=*No* 1=*Yes*)**	[__]
**Where was your baby kept after delivery**<br>Roomed in with you…… ……..1<br>Nursery………………………...2	[__]

FACTORS AFFECTING INITIATION OF BREASTFEEDING IN A TERTIARY HOSPITAL IN GHANA

Mother Baby Unit………………3
**Reason baby was sent to the MBU** (*write below*)
**Gender of child** **(0=***Female* **1=***Male***)** [__]
**Birth weight of baby** [__][__].[__] kg
**Apgar scores of baby** First minute…………………………….……….[__][__]<br>Fifth minute ……………………………..………[__][__]
**SECTION C: Obstetric History**
**Number of antenatal attendances**…………………[__][__]
**Any medical condition in pregnancy** **(0=***No* **1**=*Yes*) [__]
**If answer to question above is yes. Please specify medical condition:**
**Parity** [__][__]
**Method of delivery** [__]<br>Spontaneous Vaginal delivery…………..**0**<br>Caesarean section……………………..…..**1**<br>Assisted vaginal delivery(vacuum)………**2**
**Immediate post-partum state of mother (***Circle the appropriate answer)*<br>Post-partum haemorrhage ……………………………**0**<br>Cervical and or vaginal tear ………….……………**1**<br>Retained placenta …………………….……………**2**<br>Pre-eclampsia /eclampsia…………………………..**3**<br>Puerperal blues/psychosis……………………………**4**<br>Well /Others ……………………………………….**5**
**Type of Anaesthesia used in C/S** **(0**=*Spinal* **1=***General***)** [__]
**SECTION D: Breastfeeding History**
**Have you ever had any breastfeeding experience previously?** [__]<br>Yes ……………………….**0**<br>No ………………………..**1**
**If the answer is yes to question above, was it successful** [__]<br>Yes ……………………….**0**

No ………………………..**1**
**Time of breastfeeding initiation after delivery** [__]<br>Within one hour 0<br>Between 2-5hrs 1<br>6 to 10hrs 2<br>After 11 to 15hrs 3<br>16 to 24 hrs 4
**If answer to question above is more than 1hour, what could be the possible cause of the delay? (circle the appropriate answers)**<br>Were you still at the theatre 0<br>Too weak to start breastfeeding 1<br>Too much pain to start breastfeeding 2<br>Baby sent to the sick newborn 3<br>Others [specify] 4
**Did your child have any food or water before you started breastfeeding** [__]<br>Yes ……………………….**0**<br>No ………………………..**1**
**If your answer above is yes give reasons prelacteals was given.**
**31.Who gave prelacteals** [__]<br>Mother ……………………………….**0**<br>Nurse …………………………………**1**<br>Other(specify)………………………...**2**
